# Effects of *Tagetes minuta* essencial oil on *Lucilia cuprina* third instar larvae

**DOI:** 10.1016/j.dib.2019.104008

**Published:** 2019-06-14

**Authors:** Amanda Chaaban, Vera Maria Carvalho Silva Santos, Carlos Eduardo Nogueira Martins, Juliana Sperotto Brum, Fabiano Cleber Bertoldi, Marcelo Beltrão Molento

**Affiliations:** a Laboratory of Parasitic Diseases, Department of Veterinary Sciences, Federal University of Parana. Curitiba, PR, Brazil; b Department of Veterinary Medicine, Catarinense Federal Institute. Araquari, SC, Brazil; c Laboratory of Veterinary Pathology, Department of Veterinary Sciences, Federal University of Parana. Curitiba, PR, Brazil; d Agricultural Research and Extension of Santa Catarina, EPAGRI. Itajai, SC, Brazil; e National Institute of Science and Technology, INCT-Livestock. Belo Horizonte, MG, Brazil

**Keywords:** Cuticle damage, Natural product, Ecofriendly, Blowfly

## Abstract

The activity of *Tagetes minuta* essential oil (TMEO) was tested against third instar larvae (L3) of the Australian blowfly *Lucilia cuprina*. We have determined the potential of the *T. minuta* EO as a new biopesticide candidate. To test this, groups of 20 L3 were placed on filter paper impregnated with ranging concentrations (from 0.19 to 6.36 μL/cm^2^) of TMEO, solubilized in acetone. Data show in this article is related to research article “Tissue damage and cytotoxic effects of *Tagetes minuta* essential oil against *Lucilia cuprina*” Chaaban et al., 2019. Thus, data of cuticle damage, color changes in L3 body and decrease in L3 motility were recorded 24 and 48 h after TMEO contact.

**Table:** Specifications table

Subject area	*Parasitology*
More specific subject area	*Entomology*
Type of data	*Movies*
How data was acquired	*Microscope stereoscopy*
Data format	*Raw data collection and analysis*
Experimental factors	*Fresh aerial parts of the plants (leaves, flowers and stems) were homogenized and the essential oil was extracted by hydrodistillation for 4 h in a Clevenger apparatus. Biological assays on Lucilia cuprina performed as described in the companion paper**[1]**.*
Experimental features	*Essential oil extraction and chemical characterization.* *Establishment of Lucilia cuprina colonies; and biological assays on laboratory conditions (27± 2 °C and 70% relative humidity).* *Contact tests using filter paper impregnated with Tagetes minuta essential oil.* *Cuticular damage and larvae motility were reported.*
Data source location	*Araquari, Santa Catarina, Brazil; 26°23′ 33.6691″ S and 48° 44′ 18.3336″ W.*
Data accessibility	*Data is displayed within this article.*
Related research article	*This Data in Brief article is submitted as a companion paper to:* *Chaaban, A., Santos, V.M.C., Martins, C.E.N., Brum, J.S., Bertoldi, F.C., Molento, M.B. Tissue damage and cytotoxic effects of Tagetes minuta essential oil against Lucilia cuprina. Experimental Parasitology**[1]*

**Table:** 

**Value of the data** • Development of a potential new biopesticide against blowfly. • Contact activity of the essential oil of Tagetes minuta “marigold” over Lucilia cuprina larvae. • Determination of time-dependent damage of Lucilia cuprina larvae treated with Tagetes minuta essential oil.

## 1 Data

The data displayed in this data article involve the experimental data from the effects on cuticle damage of *Lucilia cuprina* third instar larvae, treated with different concentrations of *Tagetes minuta* essential oil [1]. The larvae of the control group, using acetone as solvent, showed no cuticle alterations after 6, 24 or 48 h of contact (Video 1–3). The essential oil from *T. minuta* showed no toxicity at 6 h of treatment (Video 4), however, a time-dependent mortality was demonstrated after 24 and 48 h of exposure. Morphologic changes in mature larvae, such as cuticle softening and dryness, color changes, as well as reduction of their motility were observed (Video 5–7).

The following are the supplementary data related to this article:

1

2

3

4

5

6

7

**Video 1.** Third instar larvae (L3) of *Lucilia cuprina*. Control group (only acetone) after 6 h of solvent exposure. Note: larvae cuticle is intact and normal motility.

**Video 2.** Third instar larvae (L3) of *Lucilia cuprina*. Control group (only acetone) after 24 h of solvent exposure. Note: larvae cuticle is intact and normal motility.

**Video 3.** Third instar larvae (mature larvae) of *Lucilia cuprina*. Control group (only acetone) after 48 h of solvent exposure. Note: larvae cuticle is intact and the formation of normal pupae.

**Video 4.** Third instar larvae (L3) of *Lucilia cuprina*. Group treated with 1.59 μL/cm^2^ (10%) of *Tagetes minuta* essential oil after 6 h of exposure. Note: normal motility.

**Video 5.** Third instar larvae (L3) of *Lucilia cuprina*. Group treated with 1.59 μlLcm^2^ (10%) of *Tagetes minuta* essential oil after 24 h of exposure. Note: the accentuated decrease in larval motility and damage to the cuticle.

**Video 6.** Third instar larvae (L3) of *Lucilia cuprina*. Group treated with 1.59 μL/cm^2^ (10%) of *Tagetes minuta* essential oil after 48 h of exposure. Note: the complete loss of larval motility, death of the organisms and accentuated cuticle damage.

**Video 7.** Third instar larvae (L3) of *Lucilia cuprina*. a) Control group (only acetone) after 24 h of exposure; b) Group treated with 1.59 μL/cm^2^ (10%) of *Tagetes minuta* essential oil after 6 h of exposure; c) Group treated with 1.59 μL/cm^2^ (10%) of T. minuta essential oil after 24 h of exposure; d) Group treated with 1.59 μL/cm^2^ (10%) of T. minuta essential oil after 48 h of exposure.

## 2 Experimental design, materials and methods

### 2.1 Plant material, essential oil extraction and chemical characterization

*Tagetes minuta* species used in the biological assays against *L. cuprina* was grown in the Medical Plants Unit of the Catarinense Federal Institute (IFC), Araquari, Brazil. Fresh aerial parts of the plants were homogenized and the EO was extracted by hydrodistillation for 4 h in a Clevenger apparatus. The essential oil was analyzed by gas chromatography coupled with a mass spectrometric detector, and mass spectra were compared with the database of the NIST library [2]. Details about this issue are described in the companion paper [1].

### 2.2 Establishment of Lucilia cuprina colonies and larval toxicity

Data of establishment of stock colonies, maintenance, mass reproduction and the protocol for the biological tests are mentioned in the companion paper [1]. The toxicity of *T. minuta* over L3 of *L. cuprina* was performed using groups of 20 L3, placed on filter paper and impregnated with a range of concentrations (0.19–6.36 μL/cm^2^). The L3 were put into glass vials containing a filter paper (12.56 cm^2^) impregnated with 0.2 mL of TMEO solutions, solubilized in acetone. The *T. minuta* toxicity was evaluated by observing L3 mortality at 6, 24 and 48 h after contact [3]. Total larval mortality (LM) was calculated [3], [4] as follows:

LM = (total died larvae x 100)/total tested larvae.

The damages were measured by macroscopic biomarker changes and microscopic lesions using histological sections. However, results of lethal doses and physiological parameters in L3 treated with TMEO can be observed in the companion paper [1].

## Conflict of interest

The authors declare that they have no known competing financial interest or personal relationship that could have appeared to influence the work reported in this paper.
